# Functional recovery from eccentric injury is maintained in sarcopenic mouse muscle

**DOI:** 10.1002/rco2.33

**Published:** 2021-06-02

**Authors:** Ana P. Valencia, Ashton T. Samuelson, Rudolph Stuppard, David J. Marcinek

**Affiliations:** Department of Radiology, School of Medicine, University of Washington, Brotman 140, 850 Republican St., Seattle, WA 98109, USA

**Keywords:** Atrophy, Fatigue, Force, Exercise-induced damage, Regeneration, Skeletal muscle

## Abstract

**Background:**

Eccentric contractions induce muscle damage (EIMD) that compromises muscle function. Poor recovery from EIMD has been suggested to be a contributor to the decline in muscle function evident in sarcopenia, but it is unclear which aspects of muscle function are more susceptible to disruption by EIMD in old versus young muscle. The purpose of this study was to determine the extent of impairment in contractile function (force, fatigue, tetanus and twitch kinetics) during the recovery from EIMD in very old (VO) mice compared to young adult (YA).

**Methods:**

Male CB6F1 were obtained from National Institure of Aging colony. VO mice were 29–31 months of age, and YA mice were 7–9 months of age. The plantarflexor muscles were subjected to 20 eccentric contractions *in vivo* to induce injury (EIMD). Changes in tetanic force and kinetics were assessed before EIMD, immediately after EIMD and 3 days after EIMD (3dEIMD). Force–frequency and rates of fatigue were assessed 3d-EIMD and compared with baseline. Histological analysis was conducted in injured and non-injured contralateral gastrocnemius muscle.

**Results:**

There was a greater loss in isometric tetanic force immediately following EIMD in VO compared with YA (−31.6% ± 10.4 vs. −21.7% ± 6.0, *P* < 0.05). At 3d-EIMD, the rate of contraction of tetanus began to recover in VO, but not in YA (20.8% vs. −6.8%, *P* < 0.05), whereas the extent of recovery of force tended to be greater in VO than YA (39.3% vs. 17.1%, *P* = 0.08) when compared with tetanic function immediately after injury. Compared with function pre-injury (baseline), VO and YA had similar deficits in tetanic force (−7.3% ± 5.3 vs. −9.2% ± 6.0, respectively) and kinetics at Day 3. Twitch kinetics (rate of relaxation) recovered faster in VO compared with YA. The rate of muscle fatigue was similar to baseline values, with VO continuing to be more fatigue resistant than YA 3d-EIMD. There were no detectable differences in muscle mass or myofibre cross-sectional area despite continued deficits in force following EIMD in either age group.

**Conclusions:**

Despite clear functional deficits and greater susceptibility to injury, aged sarcopenic muscle exhibited a similar ability to recover contractile function to younger muscle following EIMD. In addition, neither age group showed accelerated muscle fatigue in the recovery phase after EIMD; thus, sarcopenic mouse muscles do not appear to be more susceptible to long-term functional impairment than young healthy muscles.

## Introduction

The progressive loss of muscle function and mass with age, known as sarcopenia, affects millions of Americans and people worldwide. Sarcopenia is a leading cause for falls and reduced quality of life in the elderly, and currently, there are no effective therapies to prevent it. The most recommended therapy thus far is resistance training, which can delay the loss of muscle mass and improve function in people with sarcopenia.^1^ Although the capacity to exercise is diminished in sarcopenic individuals, it is imperative to find therapies that maximize muscle function that avoid the risk of injury. For many years, eccentric contractions have been known to induce muscle damage, and it has been suggested that eccentric induced muscle damage (EIMD) could worsen function of sarcopenic muscle.^2,3^ More recently, however, eccentric training has gained attention as means to improve muscle function and metabolic parameters in the elderly.^4–7^ These differing views require clarification on how EIMD affects performance of sarcopenic muscle.

Eccentric contractions are induced when the muscle is actively lengthened. Eccentric strength is necessary for healthy locomotion, and it appears to be better preserved than isometric and concentric force in older individuals.^8^ Unlike concentric and isometric contractions, eccentric contractions can also induce damage by disrupting sarcomere structure, altering the neuromuscular junction and causing membrane damage, leading to calcium influx and a cascade of stressors.^9–11^ Various studies show that aged skeletal muscle is more susceptible to injury and is slower to recover than young by measuring outcomes such as sarcomere disruption,^2^ inflammation^12^ and changes in force.^13,14^ Muscle strength is considered the best assessment of the extent of injury as it is quantifiable indicator of overall functional capacity of muscle.^15,16^ In addition to force, the kinetics and susceptibility to fatigue are functional aspects of muscle that are not commonly documented in the context of EIMD. We tested muscle performance of young and very old (VO) mice before, during and after EIMD using *in vivo* electrical stimulation of the ankle plantarflexors. The purpose of this study was to determine which aspects of contractile function (force, fatigue, tetanus and twitch kinetics) of skeletal muscle are more impaired and slower to recover after EIMD in VO mice compared with young adult (YA).

## Methods

### Animals

This study was approved by the Institutional Animal Care and Use Committee of the University of Washington. Male CB6F1 mice were purchased from the National Institute on Aging (NIA). YA mice were between 7 and 9 months of age, and VO mice were between 29 and 31 months of age. All mice were exposed to a 14-h light:10-h dark cycle in a fixed-temperature environment of 72°F and 30–70% humidity with free access to water and standard rodent chow (Formulab Diet 5008C33, Richmond, IN) prior to experiments.

### In vivo muscle force and fatigue

*In vivo* measurement of ankle plantarflexor function has been previously described^17^ using an Aurora Scientific 305C servomotor (Aurora, Ontario, Canada). Each mouse was anaesthetized with isoflurane (4% for induction and ~2% for maintenance) and laid on its side on a temperature-controlled platform maintained at 37°C. The right knee was clamped in place, and the foot was secured to a footplate with the ankle positioned at 90°. The tibial nerve was stimulated at an optimal voltage (1–5 V) using percutaneous electrodes near the popliteal fossa using a Grass Instruments S88X stimulator (Astro-Med, Inc., West Warwick, RI, USA). Force–frequency involved stimulating the muscle at frequencies from 10 to 200 Hz. After 2 min of rest, fatigue was induced by tetanus at 100 Hz (~90% of maximal force) every 5 s for 120 contractions. To ensure that the loss in force was due to fatigue, we verified a recovery of force with a tetanus at 1 and 5 min after fatigue protocol. Force–frequency and fatigue were tested at baseline and 3 days after EIMD. Analyses of muscle contractions were carried out using DMA software (Aurora, ON) to quantify force, average rate of contraction and average rate of relaxation. The function for average rate of contraction and relaxation measures the slope of the linear portion (33%–66% of maximum force) of the rise or decline in force, respectively, for each contraction.

### In vivo muscle injury (eccentric induced muscle damage)

We modified our protocol based on what has been used by previous groups.^18,19^ Muscle injury to the plantarflexors was induced by 20 eccentric contractions in YA and VO mice. The eccentric contractions were initiated by stimulating the tibial nerve at 150 Hz for 200 ms using the same set-up used for baseline measures. For the first 100 ms, the ankle remained at neutral position for an isometric contraction; for the next 50 ms, the ankle joint was rotated 40° towards dorsiflexion at an angular velocity of 800°/s to induce an eccentric contraction. The next 100 ms the foot was passively rotated back to the starting position at 133°/s. Each eccentric contraction was repeated every 1.5 min. We measured maximal isometric force and kinetics 5 min after eccentric contractions (postinjury) with the ankle placed at 90° and stimulating the tibial nerve for 200 ms at 150 and 180 Hz. The maximal value out of the two contractions was considered to be maximal force. To make sure that loss of force would be due to mechanical injury rather than fatigue, we also repeated the same protocol using the same stimulation protocol without lengthening of muscle in a separate experiment, and we found minimal loss of force (Supporting Information, Data S1).

### Histology

Twenty-four hours after the final muscle testing, mice were euthanized through cervical dislocation while under inhalation anaesthesia with 2% isoflurane. The gastrocnemius and soleus were carefully dissected from both legs and weighed. The gastrocnemius was frozen in liquid nitrogen-cooled isopentane and stored at −80 until cryosectioning. The 10-μm cross sections of the gastrocnemius belly from injured and non-injured legs from YA (*n* = 3) and VO (*n* = 4) were cut using a Leica CM1950 cryostat (Buffalo Grove, IL). Sections were stained with haematoxylin and eosin (H&E) and imaged at 20× magnification. Image J was used to calculate Feret diameter of individual myofibres (>150 fibres per sample) and to quantify centrally nucleated fibres (CNFs) (>350 fibres per muscle sample).

### Statistical analysis

Data presentation and analysis were performed using GraphPad Prism 6 software (La Jolla, CA, USA). To determine differences between the two age groups, unpaired two-tailed *t*-tests were performed. To assess the differences in recovery between VO and YA, the recovery was expressed as a percentage of baseline or post-injury values, which were then compared using an unpaired two-tailed *t*-test between age groups. To determine differences between injured and non-injured leg, a paired two-tailed *t*-test was performed. The results illustrated in tables and bar graphs are expressed as mean ± standard deviation (SD). The illustrated curves for force, fatigue and kinetics are expressed as mean with or without standard error. Significant differences were reported when *P* < 0.05.

## Results

### Very old mice lost more isometric contractile function than young adult following a series of eccentric contractions to induce eccentric induced muscle damage

**Figure 1:**
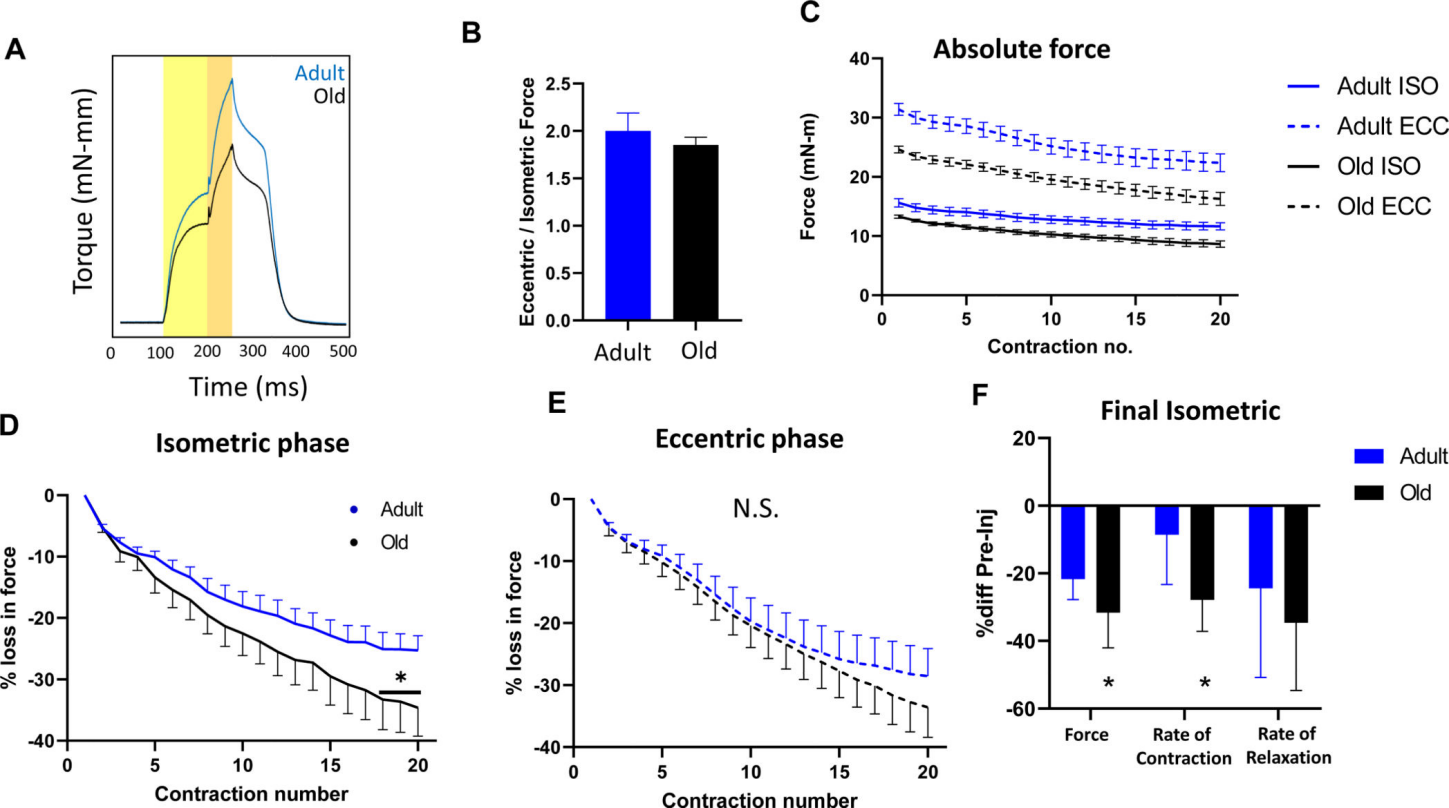
*(A)* Representative tracing of *in vivo* eccentric contraction of plantarflexors of young adult (blue) and old (black) mice. The muscle reached a maximal isometric contraction during the first 100 ms of electric stimulation (yellow highlight); the ankle was rotated towards dorsiflexion to induce an eccentric contraction (orange highlight). *(B)* The ratio of force produced in the eccentric phase to the isometric phase of the first contraction. *(C)* The absolute forces in the eccentric (dashed) and isometric (solid) phases of the contractions throughout the 20 contractions to induce EIMD. The percent loss in force of the isometric *(D)* and eccentric *(E)* phase throughout the EIMD protocol. Maximal isometric contractions were induced before EIMD protocol and following 5 min of rest. *(D)* The difference in force and kinetics was greater in old versus young adult, *n* = 9–10. *Significantly different to young adult (*t*-test, *P* < 0.05). Values expressed as mean ± SD for bar graphs and mean ± SE for line graphs.

To induce EIMD, the plantarflexors of VO and YA were subjected to lengthening contractions superimposed to maximal isometric contractions (Figure 1A). Absolute force of the initial contraction was lower in the VO compared with YA for both the eccentric and isometric phase, and the ratio of eccentric to isometric force was not significantly different between age groups (Figure 1B). Force by VO muscle remained lower than YA in the isometric and eccentric phase throughout the 20 contractions (Figure 1C). The loss of force in the isometric phase of the contraction was greater in VO compared with YA, but the loss of force in the eccentric phase was not different between age groups (Figure 1D, E). To assess the extent of injury, the force and kinetics of maximal isometric tetani were compared pre-injury and 5 min post-injury. VO had a greater loss in maximal force and rate of contraction compared with adult following EIMD (*P* < 0.05) (Figure 1F).

### Recovery of force and kinetics 3 days after eccentric induced muscle damage is similar in very old mice compared with young adult

**Figure 2:**
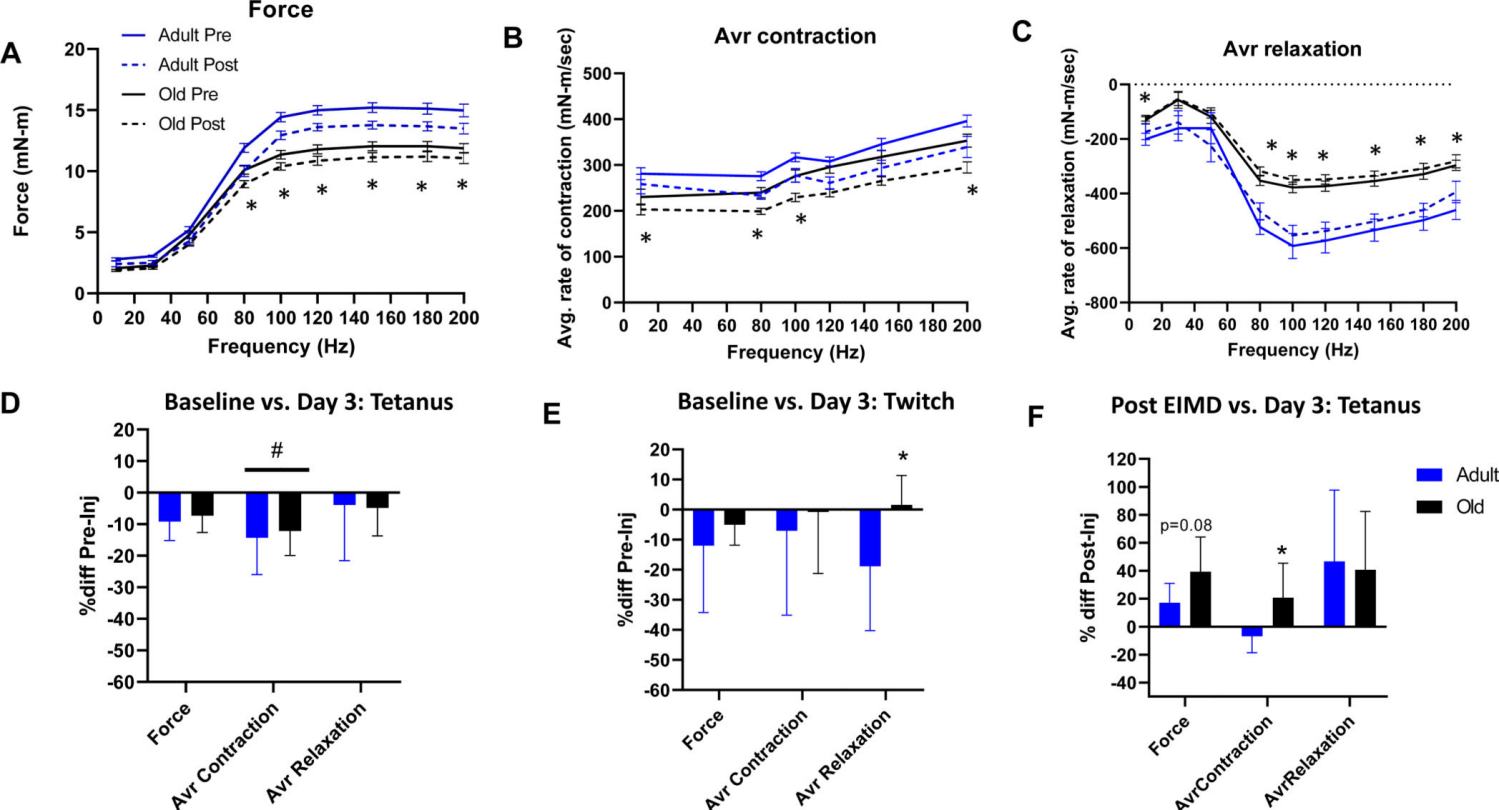
Force–frequency curves of plantarflexors were compared before (solid) and 3 days after EIMD (dashed) in young adult (blue) and old (black). *(A–C)* For each isometric contraction force, rate of contraction and rate of relaxation were recorded. *(D, E)* Compared with baseline values, young adult and old mice had similar deficits in force and kinetics for 150 Hz tetanus but not twitch. *(F)* Compared with the final tetanus immediately after EIMD, maximal isometric force and kinetics improved 3 days after EIMD, particularly in the old, *n* = 6–8. *Significantly different to young adult (*P* < 0.05). ^#^Significantly different to deficits in force and rate of relaxation (one-way ANOVA, *P* < 0.05). Values expressed as mean ± SD for bar graphs and mean ± SE for line graphs.

We compared force–frequency curves at baseline and 3 days after EIMD. At baseline, absolute force, rate of contraction and rate of relaxation remained lower and slower in the VO compared with YA for most frequencies with the exception of absolute force at low frequencies (10–50 Hz), where there were no differences between age groups (Figure 2A–C). Recovery of muscle function is indicated by the per cent difference from the baseline values (Figure 2D, E) and difference from the post-injury values and (Figure 2F). By Day 3, both VO and YA showed similar deficiencies in tetanic function compared with baseline, with rate of contraction being the parameter most significantly depressed relative to baseline (Figure 2D). VO showed better recovery of twitch function, specifically rate of relaxation (Figure 2E). The amount of isometric function regained 3 days after injury was greater in the VO compared with YA, particularly rate of contraction that improved 20.8% ± 24.7 in VO, but continued to decline mildly in YA by −6.8% ± 11.8 relative to the rate immediately post-injury (Figure 2F). By Day 3, isometric force improved 39.3% ± 24.8 for VO and 17.0% ± 14 for YA relative to immediately post-injury, but age differences did not reach statistical significance (*P* = 0.08).

### Fatigue is not affected 3 days after eccentric induced muscle damage in either age group

**Figure 3:**
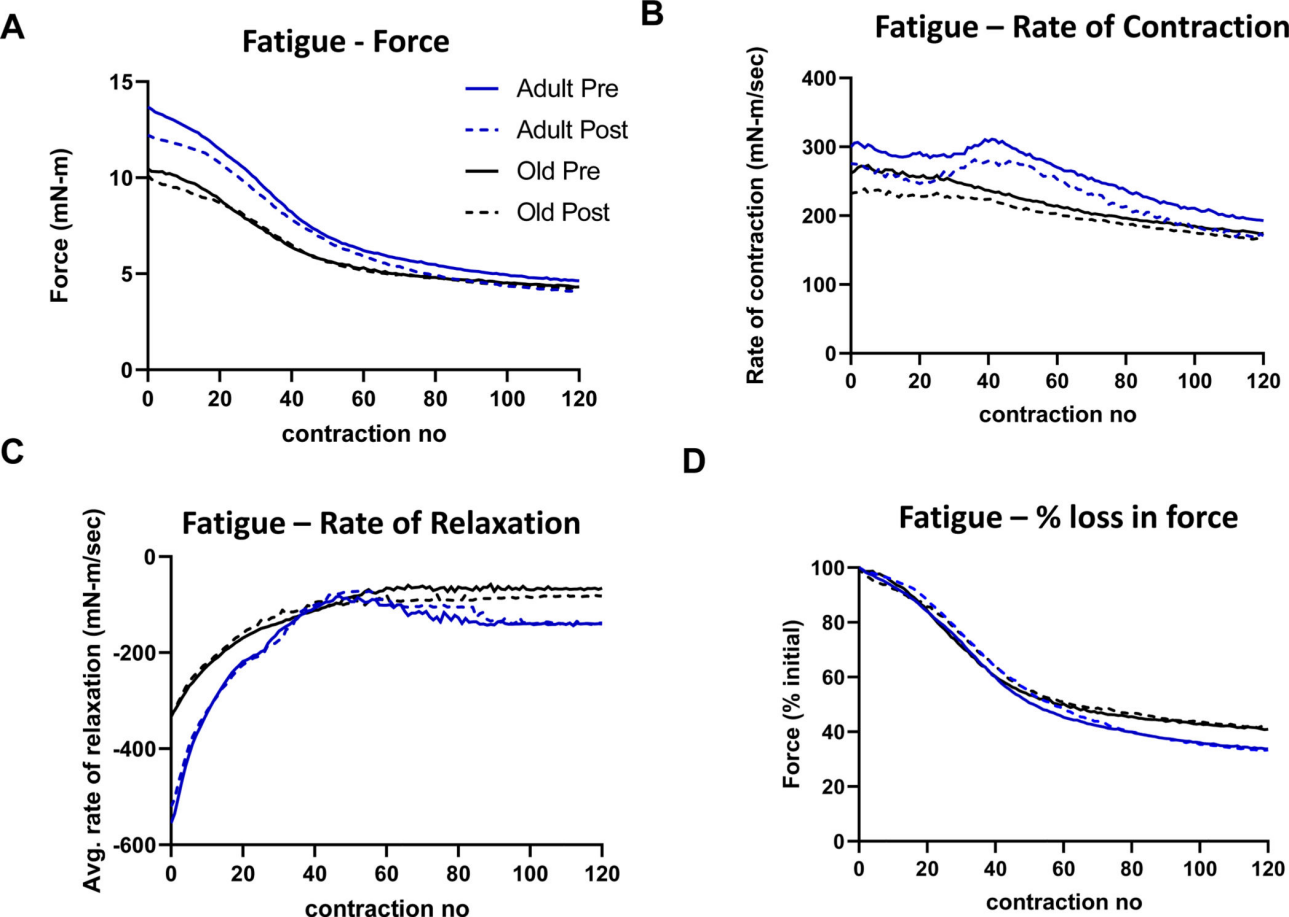
Muscle fatigue of plantarflexors was assessed *in vivo* and compared before (solid) and 3 days after EIMD (dashed) in young adult (blue) and old (black). *(A–C)* For each isometric contraction force, rate of contraction and rate of contraction were recorded. *(D)* Compared with the initial isometric contraction, the decline in force was similar at baseline and 3 days after EIMD for both age groups, *n* = 6. Only the mean values are expressed.

We then asked if rate of fatigue would be affected 3 days following EIMD in VO compared with YA. Whereas absolute force, rate of contraction and rate of relaxation remain lower and slower in old muscle (Figure 3A–C), the rate of fatigue in the recovering muscle is very similar 3 days following EIMD to baseline, with VO maintaining more fatigue resistance pre- and post-injury (41.0% ± 4.9 and 41.6% ± 10.0, respectively) than YA (33.7% ± 2.9 and 33.4% ± 3.5) (Figure 3D).

### Muscle mass and fibre area are not affected 3days after eccentric induced muscle damage in either age group

**Figure 4:**
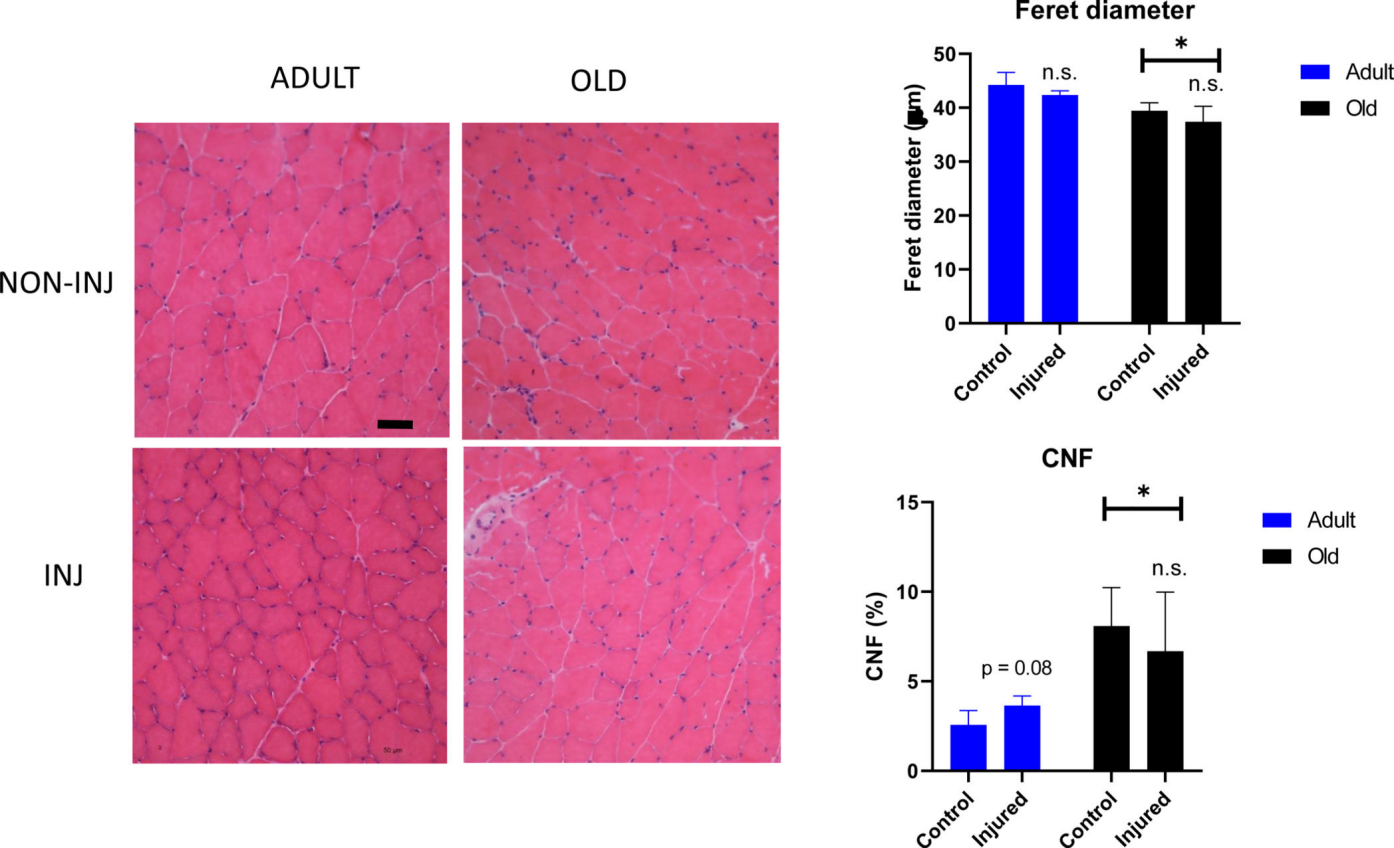
Histological analysis of gastrocnemius muscles 4 days after EIMD compared with contralateral uninjured control muscle. *(A)* Representative images of H&E-stained sections of the muscle belly used to measure Feret diameter *(B)* and quantify centrally nucleated fibres *(C)*. *Significantly different to young adult (*t*-test, *P* < 0.05). *n.s*. represents no significant difference between injured and non-injured muscle. *n* = 3–4. Values expressed as mean ± SD. Scale bar represents 50 μm.

Muscle weights of two plantarflexor muscles, the gastrocnemius and soleus, were compared between the injured leg and the contralateral uninjured leg (Table 1). Although there were clear differences in weights and myofibre area between the VO and YA, no differences were detected between the injured and non-injured contralateral muscle mass or myofibre area in VO or YA (Figure 4A, B). VO had a greater proportion of CNFs compared with YA. There was a trend for more CNFs in injured muscle compared with contralateral control in YA (*P* = 0.08), but no differences were detected between injured and non-injured leg in VO (Figure 4C).

## Discussion

Contrary to our initial hypothesis, aged mice with sarcopenia recover muscle contractile function just as well as YA mice following EIMD, despite being more susceptible to eccentric muscle injury. We evaluated force and kinetics of muscle contraction *in vivo* in the same mice immediately post-injury and at Day 3 of recovery. Muscle force and kinetics were impaired following a series of eccentric contractions in both YA and aged mice, but the loss of tetanic force and rate of contraction was greatest in aged mice. Given the greater loss of contractile function in aged mice, it was surprising to see similar force deficits between age groups at Day 3 of recovery. Several studies have shown that muscle from aged rodents take longer to recover force from EIMD,^14,20,21^ but the inconsistencies may lie on the severity of injury, time point studied and methods used. One study that reported impaired recovery of force 2 months after EIMD in plantarflexors of old mice (~70%) compared with young (~98%) used an *in situ* injury protocol that led to ~72% force deficit 3 days after injury in both age groups.^21^ Similar findings have been reported when the muscle is tested *in situ*,^14,20^ which requires exposure of the tendons that can induce additional injury.^22^ Therefore, the results from this present study may be dependent on the specific conditions of the EIMD protocol that leads to a substantial recovery of force by Day 3 post-injury (~85%), and using an *in vivo* method to induce muscle injury and test muscle function.

Injury following EIMD has been previously described to occur in two phases. The first phase is the loss of force that occurs immediately after EIMD due to mechanical injury, and the second phase occurs 2–3 days after EIMD when regeneration is active and function partially recovers.^16,23,24^ The loss of force in Phase 1 is partially attributed to disruption in sarcomeric structure, sarcolemma injury, E–C coupling impairment and reduction in cytoskeletal and contractile proteins.^25–28^ These parameters depend on the magnitude of the active strain as well as force of contraction, with maximal activation and strain resulting in a greater degree of injury.^9,29,30^ The loss in force following eccentric contractions can happen rapidly and early during the bout of contractions.^9,25,30^ We found that the loss in isometric force with maximal eccentric contractions was rapid and greater in aged mice. Immediately following EIMD, the declines in force and contractile kinetics (30%–35%) were similar in the aged mice. In contrast, younger mice lost about 25% of force but significantly less in rate of contraction (15%). It can take up to 3 days for calcium release and reuptake by the sarcoplasmic reticulum (SR) to be affected following EIMD using younger mice,^31^ and based on our observations of the immediate decline in muscle kinetics in aged muscle, it is possible that SR disruption happens much sooner in older mice. Although we cannot fully attribute the changes in tetanus kinetics to changes in E–C coupling, the speed of contraction is dependent on it, and our results could indicate that muscle kinetics are protected in young muscle immediately after EIMD but not in old.

Tissue regeneration is active within a few days after injury during the second phase of EIMD. Inflammation, fibre swelling, edema, joint stiffness and delayed onset of muscle soreness are apparent. Given that old muscle lost more force after EIMD, and ageing is often characterized by impaired regeneration, we expected for muscle from old mice to perform more poorly than muscle from young 3 days after injury. Unlike other studies, our assessment of recovery was strictly functional, focused on tetanic force, muscle kinetics and fatigue. Others have utilized different markers of muscle injury such as sarcomere disruption, plasma creatine kinase (CK) or inflammation that also led to inconsistent conclusions.^2,32,33^ Although our assessment of contractile function did not detect differences between age groups at Day 3 following EIMD, it is still possible for age differences to be detectable at later time points. A study focused on the recovery phase after EIMD, kept force loss with EIMD in the extensor digitorum longus (EDL) similar between young and old and found that by Day 3 muscle force was still similar in both age groups though still ~60% weaker than the control muscle. It was not until 28 days after injury where force recovery was different between young and old, where young had fully recovered and old still had a force deficit of ~15%.^20^ However, the severity of injury of this study is greater than we report here because both age groups in the present study had a 10% deficit in force by Day 3.

The reduction in regenerative capacity, often associated with age, does not consistently influence functional recovery after EIMD. Studies performed on muscle regeneration often rely on injury models that are more severe than EIMD. For instance, the injection of cardiotoxin into the muscle is a common tool to induce muscle damage, and following a week of cardiotoxin injury, the muscle is only able to produce up to 50% of its original force.^34^ In order to challenge muscle regeneration, one has to maximize the severity of injury and degree of structural damage. Old animals often show delays in muscle recovery using these methods,^35^ though not consistently.^36^ One of the factors that contributes to impaired regeneration with ageing is the decrease in satellite cell number or function. Inducible depletion of satellite cells in adult mice leads to increased fibrosis and impaired regenerative capacity after cardiotoxin injury. However, after EIMD mice lacking satellite cell proliferation through γ-irradiation had similar functional deficits to control up to Day 7,^37^ suggesting that the improvement of force that occurs 0–7 days after EIMD is not dependent on satellite cells. In addition, the force deficits and responsiveness of satellite cells at Days 2 and 7 after EIMD were not different between young and older adults.^38^

We also tested muscle fatigue, and neither young nor old muscle was more susceptible to fatigue during the recovery phase following EIMD. Muscle fatigue can lead to changes in motor control and increase the risk of falls in elderly. More is known about the effects of fatigue on susceptibility to EIMD. Muscle fatigue prior to EIMD has been shown to either be protective or have no influence on force loss after EIMD.^39,40^ Incorporating muscle fatigue into an EIMD protocol, however, exacerbates the loss of force.^10^ Here, we show that muscle fatigue 3 days after EIMD is not affected by EIMD; thus, changes in locomotion 3 days after injury may be more representative of impaired force and soreness, rather than an increase in muscle fatigue.

In human subjects, reports of damage, recovery and susceptibility to EIMD with age are inconsistent. Muscle damage immediately after exercising in an eccentric cycle ergometer was greater in the old compared with young, where 95.7% of fibres from biopsy samples of vastus lateralis showed disruptions such as focal damage, Z-band streaming and spreading, dissolution of the Z-band and fragmented SR, compared with 5%–10% in YA.^2,41^ However, after downhill running, young men experienced a greater rise in CK levels compared with older participants,^32^ and adult subjects lost more force immediately after eccentric contractions of elbow flexors compared with older subjects.^42,43^ Another recent study found no age differences in the loss of force and recovery of force following EIMD in plantarflexors of human subjects.^38^ Contradictory conclusions from human studies are likely due to a variety of factors including muscle groups tested, method of injury, measurement of injury and training status and sex of population studied.^44^ When these confounders were controlled for in human subjects, age was not a predictor of recovery of force production, but myonuclear and capillary numbers and habitual physical activity were associated with recovery from injury.^38^

We found that 3 days after injury, the major functional deficits were in tetanic rate of contraction and specific force in both age groups. Calcium release has been shown to decline 20% in injured EDL at this time point.^31^ Up to 50%–70% of the deficits in force 0–5 days after injury is attributed to E–C uncoupling and the remaining deficit as a result of physical disruption to force-bearing elements.^26^ E–C coupling refers to the sequence of events starting from passage of the action potential across the sarcolemmal membrane down to the T-tubule and ends with the release of calcium from the SR to be used for muscle contraction.^45^ Failure at the level of the sarcolemma has been shown in mouse models of Duchenne muscular dystrophy and Niemann-Pick Type A/B disease,^28,46^ but the ability to conduct action potential through the sarcolemma is not usually affected in injured fibres from wild-type mice.^47^ In fact, resealing of the membrane can occur within just 2 min after injury.^48^ Even human studies did not detect injury to the sarcolemma after downhill running, suggesting that it is not the main contributor to force loss after EIMD.^33^ Instead, the site of E–C failure may be at the level of the voltage sensors on the T-tubule and ryanodine receptor (RyR) on the SR that later results in impaired calcium release and uptake by the SR.^26,31,45^ The disrupted communication appears to be partly due to immediate degradation of proteins known as junctophilins, which tether the T-tubule membrane with the SR membrane, as well as FKBP12, which binds to subunits on the RyR and stabilizes the closed state of the SR Ca^+^ channel.^49,50^ The change in junctophilin expression after EIMD is strongly associated with the amount of force lost in the muscle immediately after injury,^50^ but their expression recovers to baseline levels by Day 3 after EIMD, suggesting that they may play a role in the rapid recovery of E–C function after EIMD. It is unclear if the failure site is the same in ageing muscle. Recovery of E–C coupling can occur faster than the process of muscle regeneration, and given the quick recovery of force by old mice, we can infer that most of that recovery was due to improvements in E–C coupling. That would also explain why twitch force and kinetics were mostly recovered by Day 3.

Ageing sarcopenic muscle can maintain remarkable plasticity in response to stressors. For instance, a histological analysis of muscle grafts transplanted into young and geriatric mice reported similarities in myogenesis between geriatric and young mice by comparing the density of newly formed myotubes.^51^ Older adults also have shown similar activation to angiogenic and myogenic processes to younger adults after EIMD of the plantarflexors.^38^ A recent study using RNA sequencing compared the molecular networks that were differentially activated by age in response to eccentric contractions.^52^ They found that both young and old participants had similar declines in strength following eccentric contractions and found for M12 cell-adhesion molecule to be upregulated by eccentric contractions and positively correlated with the decline in strength in both age groups. They also found that eccentric contractions increased the myeloid cell differentiation-related molecular network in old muscle but not in young, suggesting that this network may be a unique response to ageing muscle that allows it to recover from EIMD. The plasticity of ageing muscle, especially following EIMD, is consistent with the observed benefits of eccentric training in older individuals.^53,54^ A strength training programme consisting of eccentric resistance exercise three times a week led to significant improvements in strength, balance and decreased risk of falls. In addition, there are ways to mitigate the amount of damage or soreness from EIMD, such as conditioning the muscle with eccentric contractions once a week,^55^ flexibility training^56^ and decreasing the intensity of the eccentric contractions.^57^

In conclusion, despite greater impairments in isometric force and rate of contraction in aged skeletal muscle following EIMD, aged skeletal muscle exhibited remarkable recovery of contractile function 3 days after EIMD. In fact, the amount of function recovered after EIMD was greater in the old compared with young, so by Day 3 after EIMD, there were no differences in functional deficits between age groups. We also tested muscle fatigue, expecting it to worsen after EIMD particularly in the old, but found it to be similarly affected in both age groups. Contrary to our hypothesis, aged sarcopenic muscle maintains its capacity to functionally recover from EIMD.

## Supplementary Material

1 **Data S1.** Supplemental 1. Loss of isometric force of plantarflexors *in vivo* during 20 eccentric (red) and isometric (black) contractions. The muscle was stimulated via the tibial nerve for 200 ms at 150 Hz every 1.5 minutes. A final isometric contraction following 5 minutes of rest was compared to the initial isometric contraction to determine force loss.

## Figures and Tables

**Table 1:** Gastrocnemius and soleus wet weight

				Two-tailed *t*-test (age)	Two-tailed paired *t*-test (injury)	

		Adult	Old			
		
		(*n* = 7)	(*n* = 5)		Adult	Old

Body weight (g)		42.3 ± 5.0	37.9 ± 6.0	*P* = 0.061		
Gastrocnemius (mg)	NON-INJ	202.7 ± 10.8	159.4 ± 12.7	*P* < 0.01	*P* = 0.99	*P* = 0.14
	INJ	202.7 ± 14.7	157.5 ± 11.7	*P* < 0.01		
Soleus (mg)	NON-INJ	10.4 ± 1.0	7.9 ± 1.7	*P* = 0.053	*P* = 0.87	*P* = 0.92
	INJ	10.5 ± 0.9	8.6 ± 1.1	*P* < 0.05		
