# Supplementary figures and images for: Functional recovery from eccentric injury is maintained in sarcopenic mouse muscle
Source: JCSM Rapid Commun. Author manuscript; Available in PMC 2024 Jul 19. (PMC11258993; doi:10.1002/rco2.33)

# Supplemental Figure

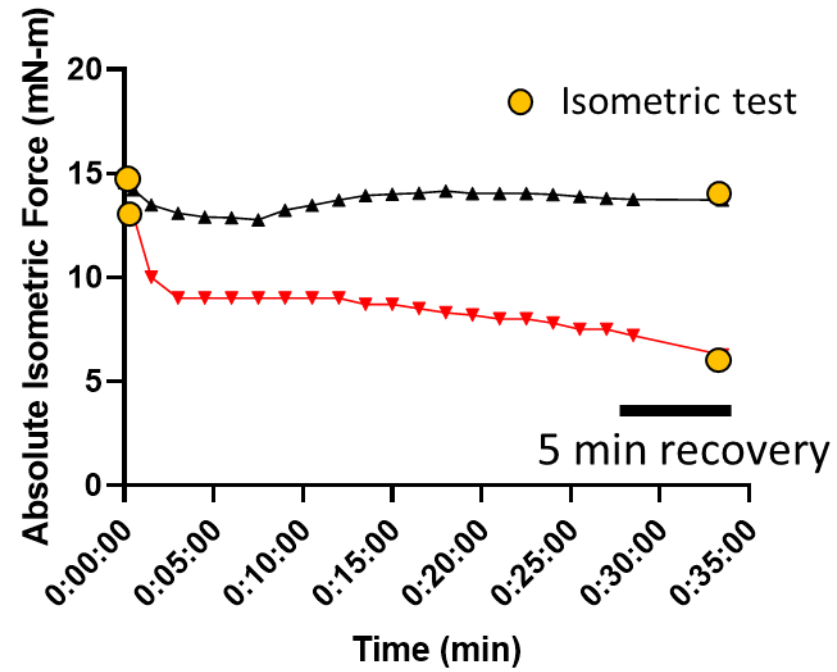

Supplement: 1 — Data S1. Supplemental 1. Loss of isometric force of plantarflexors in vivo during 20 eccentric (red) and isometric (black) contractions. The muscle was stimulated via the tibial nerve for 200 ms at 150 Hz every 1.5 minutes. A final isometric contraction following 5 minutes of rest was compared to the initial isometric contraction to determine force loss. [file NIHMS2004124-supplement-1.pdf]
